# “*What Other Information Is There?*”: Identifying Information Gaps, Perceptions and Misconceptions on COVID-19 Among Minority Ethnic Groups in the Netherlands

**DOI:** 10.3389/frhs.2022.824591

**Published:** 2022-07-08

**Authors:** Amisah Zenabu Bakuri, Daniel Antwi-Berko

**Affiliations:** ^1^Amsterdam Institute of Social Science Research, University of Amsterdam, Amsterdam, Netherlands; ^2^Center for Conflict Studies-History of International Relations, Utrecht Unviversity, Utrecht, Netherlands; ^3^Department of Basic and Applied Biology, University of Energy and Natural Resources, Sunyani, Ghana

**Keywords:** COVID-19, Ghanaian-Dutch, Surinamese-Dutch, perceptions, information gap, misconceptions

## Abstract

**Background:**

Multiple media platforms and various resources are available for information on the novel coronavirus disease (COVID-19). Identifying people's preferences is key to building public confidence and planning for successful national or regional health intervention strategies.

**Methods:**

Using exploratory mixed-methods including a short survey, interviews and participant observation, this cross-sectional study of 160 respondents from the Ghanaian-Dutch, Afro and Hindustani Surinamese-Dutch communities in Amsterdam, the Netherlands was conducted. Data collected between February to April 2021, included demographics characteristics, knowledge, opinions, preferred source of information, behavioral factors, and information gaps on COVID-19 prevention measures, responses and decision-making of respondents. Descriptive statistics and follow-up in-depth interviews were conducted to determine the relationship between respondents' demographics, information sources, and attitudes/behaviors toward COVID-19.

**Results:**

The findings of this study indicated that although many of the respondents from these communities had good knowledge on COVID-19, its modes of transmission and prevention measures, their willingness to take up initiatives and prioritize self responsibility toward their health are tied to their communal life. The respondents in this study demonstrated high value for social lives and relied on their connections with friends and families in shaping, obtaining, processing and utilizing COVID-19 information to build a sense of responsibility toward the uptake of COVID-19 prevention measures despite recent decline in number of cases.

**Conclusion:**

This sense of responsibility means their active participation and ownership of interventions to address the specific personal concerns and that of their community. However, different factors play influential roles toward the behavior choices of our respondents regarding the COVID-19 prevention.

## Introduction

Since its discovery in December 2019 in Wuhan city, the Hubei province of China, the Coronavirus disease (COVID-19) rapidly evolved into a global pandemic affecting the normal functioning of all nations, societies, and health systems (1). The COVID-19 pandemic is considered as one of the biggest global health crisis of this century and continues to impose enormous strain on individuals, communities and healthcare systems (2). As of 6th April 2021 when data collection for this study was at concluding stage, the World Health Organization (WHO) estimates showed that more than 130 million people had contracted COVID-19 with over 2.8 million reported deaths globally (3).

At the onset of the pandemic, the WHO strongly recommended countries to implement interventions to curb the rapid spread of COVID-19 by minimizing contact between infected and uninfected persons (4). So far, these measures have included mass testing, lockdowns, staying/working from home, physical distancing, self-isolation/quarantine, use of personal protection equipment (including use of face/nose masks), rigorous methods of personal hand hygiene and the rollout of various national mass vaccination (4–6). In the Netherlands, these measures included the imposition of ban on large gatherings, closure of schools and public places (5). These mitigation measures were targeted at reducing the burden of healthcare systems, rapid transmission and curb the mortality rates related to COVID-19 (4, 5, 7, 8).

These measures were necessary at the onset of the pandemic to help health systems and policymakers to adopt strategies to adequately tackle the virus. However, certain community risk perceptions and poor adherence to these preventive measures have led to an increased rate of infection as seen in Ethiopia and the Netherlands (9, 10). For instance, a significant proportion of young people particularly university students assumed that the disease only affects the elderly and people with underlying medical conditions (11, 12). Others have considered COVID-19 as hoarse and assume perhaps it is a political conspiracy to control people (13, 14). These happenings epitomized the level of misconception, laxity of government and public health policies, inadequate education, inadequate information, and misperceptions toward COVID-19 (15). The continuous growth of these negative ideas and behaviors toward the global fight against COVID-19 remains a great concern that needs to be addressed.

COVID-19 continuous to be regarded as a major public health threat globally. In the Netherlands, official figures from the National Institute for Public Health and Environment [Rijksinstituut voor Volksgezondheid en Milieu (RIVM)] showed that more than 1.3 million people have been confirmed to have contracted COVID-19, with 16,629 reported deaths as of 6th April 2021 (5). The Netherlands ranks 21st worldwide, and 10th in Europe regarding the distribution burden of COVID-19 reported cases (16). To this effect, the Dutch government initiated rigorous COVID-19 testing/screening program (Test locations) in all its administrative municipalities (GGDs), with strong enforcement of curfews at some periods in attempt to halt the spread of the virus (5). Efforts to increase community awareness have also been initiated (17).

In Amsterdam, where this study was conducted, the risk of COVID-19 remains high. A recent study conducted among six (6) ethnic groups in Amsterdam revealed that the minority ethnic communities in the Zuidoost sub-district of Amsterdam had the highest COVID-19 antibody prevalence (10). Their findings also revealed that the these ethnic minority communities had the lowest numbers in terms of testing per 100 thousand residents and appeared to be the hardest hit in Amsterdam (10, 18). The minority ethnic groups that have been severely affected by the COVID-19 pandemic include the Ghanaian-Dutch, Afro Surinamese-Dutch and the Hindustani Surinamese-Dutch communities in Amsterdam (10).

People's knowledge, opinions, perception and beliefs are determinants of health behaviors (19). To this effect, there is the need for information on these communities perceptions and knowledge of the COVID-19 recommended prevention measures. However, to date, no study has been carried out to assess these communities information needs and behavioral responses toward COVID-19 mitigation measures in the study area. Therefore, this study aimed to assess the information gaps, behavioral factors, knowledge and perceptions on COVID-19 among the Ghanaian-Dutch and Surinamese-Dutch (Afro- and Hindustani) residents in Amsterdam. It is important to note that data collection for this study was collected from February to April 2021. Despite the rapid development in terms of mass vaccination, reduction in cases, severity of the disease and reduction in hospitalization, there are people who rationalize these developments as prove that COVID-19 was a hoarse, created by goverments to regulate the population. We show that the evolving situations of the pandemic reproduces and further contextualizes our understanding of the pandemic and how people respond and adapt to their changing information needs.The findings of this study will also help decision-makers and COVID-19 task forces design and inform public health communication efforts toward eradicating this pandemic or future pandemics among minority ethnic communities.

## Methodology

### Study Area

The study was conducted in the Zuidoost sub-district of the city of Amsterdam, the capital and most populous city of the Netherlands. Historically, this area has gained a reputation of high social life, an entertainment and shopping hub mostly due to its open, modern architecture and multiethnic population. Earlier report of the number of people tested for COVID-19 per 100 thousand residents revealed that the Zuidoost was one of the sub-districts with the lowest testing rate and also one of the hardest hit areas in Amsterdam (10, 18). The Zuid Oost area of the municipality is ethnically highly diverse, and often referred to as Amsterdam city's “black neighborhood” due to the settlement of African migrants (20). Official records show that the three largest ethnic groups that reside in this sub-district are people of Ghanaian descent, Afro and Hindustani Surinamese descent (21, 22).

### Study Population

Respondents in this study were selected from the Ghanian-Dutch, the Afro and Hindustani Surinamese-Dutch Communities in Amsterdam. There is a wide diversity among the Surinamese-Dutch populationin in the Netherlands. Surinamese with an African background (referred to as Afro Surinamese or “Creole” in the Dutch context) are those who trace their roots to West African, and those with a South-Asian background (referred to as “Hindustani” in the Dutch context) have their roots in North India (23). According to figures published by the Statistics Netherlands, there are 356,402 people of Surinamese origin, making up nearly 2.1% of the Dutch population (22). Available records showed that about half of the 12,184 officially registered people of Ghanaian descent in Amsterdam reside in the Bijlmermeer (popularly known as Bijlmer), a suburb of Zuid Oost (Southeast) municipality (21, 24) and they form a closely-knit community and are predominantly religious (24, 25).

### Study Design

This was a community-based cross-sectional study that applied a mixture of quantitative and qualitative methods including administration of survey, participant observation and in-depth interviews, which allowed for triangulation of the data to increase its accurateness. Data collection was conducted from 3rd February, 2021 to 30th April, 2021.

### Ethical Considerations

The Ethics Advisory Board of the Amsterdam Institute of Social Science (AISSR) at the University of Amsterdam (UvA) approved this study as part of a bigger research project. The purpose, nature, and procedures of the study were clearly explained to all potential respondents. All respondents who took part in this study understood that participation in the study was voluntary and that they could withdraw from the study at any time. To ensure the anonymity of the respondents, we have used pseudonyms and changed their occupations, and places of residence when these characteristics were not directly relevant to the analysis in this article.

### Data Collection

#### Recruitment of Study Respondents

This study included respondents aged 18 years, who were recruited through personal invitations on the streets, from churches, online social media platforms, community parks, and snowballing.

#### Structure of the Survey

Based on review of relevant literature, a standard structured survey was design and used to collect data on socio-demographics (age, gender, level of education, occupation, ethnicity and household composition), knowledge about the likely sources of contracting COVID-19, prevention measures and information gaps/needs on COVID-19 for respondents and their community.

#### In-depth Interviews

In-depth interviews (IDI) were conducted as a follow-up to the collection of the survey data when respondents indicated that they would like to be invited for further interviews. During the IDIs, the questions that generated further elaborations from the respondents were probed to investigate reasons for any discrepancies between what people said or do with data from the survey. In addition, this study explored further to understand the choices people made regarding obeying the COVID-19 mitigation measures or otherwise and the motivations behind those choices. This research technique was adopted to ensure the validity of the data on social behaviors and provide an understanding of the factors behind the choices people made. The IDIs were also used to discuss *immediate past* practices of respondents that informed their current behavior, knowledge and opinions. This allowed for the researchers to link the choices of the respondents to context and changing time.

#### Participant Observation

Participant observation was a continuous element during data collection of all the 36 in-depth interviews. Substantial amount of time was spent to follow-up or accompany our respondents to places that our study respondents frequently visited as well as popular public places that Amsterdam residents of Ghanaian or Surinamese background visited mostly for shopping or to socialize. The research team also visited some churches to observe the interaction among congregants present. Besides generating important contextual information, participant observation enabled the building of rapport with respondents and also generated conversations on respondents knowledge on COVID-19. These observations were very useful in analyzing question we asked respondents that required them to indicate frequency of an action and allowing them to bring out their lived experience and show the internal structure in their surroundings, environment or society.

### Determination of Study Variables

#### Knowledge on COVID Modes of Transmission

The composite variable for measuring knowledge about the likely sources (places) of contracting COVID-19 were listed as presence at Church/Mosque, workplace, home, funerals, weddings, public and social events, restaurants/bars, public transport, marketplaces/shops, general practitioner/dentist/pharmacy post, travel and others as identified by the respondents. Respondents who selected the median, six or more of these places were labeled as having good knowledge, from 3 to 5 as having average knowledge and 2 or below as poor knowledge (26).

#### Knowledge on COVID-19 Prevention Measures

The composite variables for measuring knowledge on COVID-19 prevention measures were listed as proper hand washing and hygiene, using a face mask, keeping 1.5m physical distance, staying home, avoiding social and public gatherings, avoid or reduce visiting friends and family and getting tested and vaccinated for COVID-19. Respondents who selected the median or above the score (six or more) of these measures were labeled as having good knowledge, from 3 to 5 as having average knowledge and 2 or below as poor knowledge (26). For each measure, a proportion of respondents who know about it were calculated as a percentage.

#### Behavioral Risk of Contracting COVID-19

This was measured using the frequency of visits to crowded places or mass gatherings that make it likely to contract COVID-19. Respondents who visited these places *Always*, were classified as at high risk, *Sometimes* and *Often* as at moderate risk and rarely or never as at low risk.

#### Information Gaps

Respondents to this survey were asked to indicate their willingness (Yes, No or maybe) to receive additional information on COVID-19 and also list the specific kind of information on COVID-19 that they and/or members from their community would like to receive. In addition, respondents were requested to state their main sources of acquiring information on COVID-19.

### Data Analysis

The data collected through printed questionnaires and Google forms were entered into excel and exported for analysis using SPSS software (SPSS Inc). The descriptive proportions of respondents who used each common source to obtain information about COVID-19 were presented in terms of number and percentage. When applicable, relevant quotations of study participants from the in-depth interviews were cited to demonstrate the point and analysis.

## Results

### Socio-Demographic Characteristics of Study Respondents

At the end of the survey, a total of 160 responses for the survey and 36 IDI were collected through face-to-face, telephone and online interviews. The tabular presentation of the sociodemographic characteristics of the study respondents have previously been published (27). In brief, there were a total of 86 (53.8%) male respondents compared to females 74 (46.2%). Unlike the Afro (*n* = 54) and Hindustani (*n* = 49) Surinamese-Dutch, there were more female respondents than the males among the 57 respondents from the Ghanaian-Dutch community. Respondents belonging to the 18–25 years age group constituted the lowest proportion while those in the 36–45 years had the highest proportion, with a median age range of all the respondents between 36–45 years. All but one of the respondents had some level of formal education ranging from primary school to doctoral degrees. The single most popular employment sector for majority of the respondents was in the hospitality/catering field 23 (14.4%), followed by the unemployed or retired category 21 (13.1%) and healthcare 21 (13.1%). A total of 29 (18.1%) respondents preferred not to answer the question regarding their employment area. The majority of respondents' households were composed of 5 or more people 43 (26.9%), followed by those that had 2 persons, 42 (26.3%) and 3 persons 27 (16.9%). There were 61 individual respondents' homes with 2 adults living together, which constituted the highest proportion (38.1%).

### Knowledge Level and Behaviors Toward COVID-19

**Table 2** shows that nearly half, 78 (49.1%) of the 160 respondents demonstrated good knowledge on the likely sources or places people could contract COVID-19. A further 45 (28.1%) also demonstrated average knowledge level while a lower proportion of respondents 37 (22.8%) showed poor knowledge level on how people could contract COVID-19. With regards to knowledge of respondents on COVID-19 prevention measures, our results revealed that a total of 86 (53.8%) respondents had good knowledge of the recommended prevention measures in the Netherlands. Additional 20 respondents (20%) demonstrated average knowledge while 42 (26.2%) respondents demonstrated a poor knowledge level on the recommended COVID-19 prevention measures (Table 1).

**Table 1:** Knowledge level and behavioral characteristics toward COVID-19 among respondents from the Ghanaian-Dutch (GD), Afro-Surinamese (ASD), and Hindustani Surinamese-Dutch (HSD) communities in Amsterdam.

**Variable**	**GD**	**ASD**	**HSD**	**Total**
**Knowledge on likely places to contract COVID-19**					
Good	28	26	24	78 (49.1%)
Average	16	17	12	45 (28.1%)
Poor	13	11	13	37 (22.8%)
**Knowledge of COVID-19 prevention measures**					
Good	30	30	26	86 (53.8%)
Average	12	10	10	32 (20.0%)
Poor	15	14	13	42 (26.2%)
**Behavioral risk of contracting COVID-19**					
High	5	6	4	15 (9.1%)
Moderate	32	30	25	87 (54.5%)
Low	20	18	20	58 (36.4%)

The results also showed that over a third of the respondents 58 (36.4%) demonstrated low behavioral risk of contracting COVID-19 as they indicated that they avoid or rarely used the public transport, or attended public and social events i.e., the church, mosque, supermarket, public transport, visit friends and family, among others. More than half 87 (54.5%) had a moderate behavioral risk of contracting COVID-19 as they often or sometimes visited some of the locations noted earlier. Only 15 (9.1%) of the respondents showed high-risk behavior, as they were always present at public and social events (Table 1). It was also observed from our fieldwork that in as much as there was lockdown, it hardly deterred people from still gathering together in large numbers. More so, the study area as indicated above had many shops and so while only essential stores were open there were still many market activities at the period. From our in-depth interviews, we noted that the top three measures respondent had difficulty adhering to included wearing a face and nose mask, avoiding visits to friends and family, and maintaining an interpersonal physical distance of 1.5 m. Analysis of our in-depth interviews from this study revealed that some respondents felt that their close relatives would not contract COVID-19 and therefore did not need to keep the 1.5 m physical distances, wear a facemask, or avoid hugging and handshakes. Regarding face and nose mask, some people struggled with having something covering their faces or their well-done make-up. “I can't breathe with a mask on”, “wearing face mask is uncomfortable”, “I can not speak well with face mask” and “I am not sure my voice and words come out clear enough”, “the face mask covers my beautiful make-up and cleans it” were common complaints.

### Information Gaps

#### Respondents Information Needs

**Figure 1:**
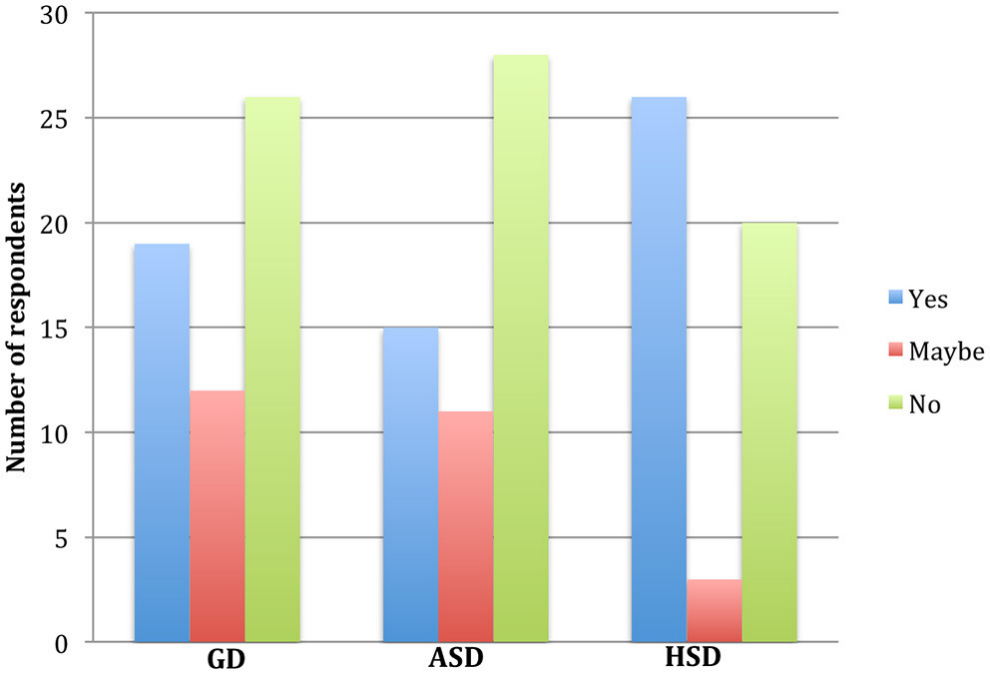
The proportion of respondents answer to the question “would you like to receive more information about COVID-19?” *The blue bars indicate a “yes”, red bars indicate a “maybe” and the green indicate a “No” responses to the question among respondents from the Ghanaian-Dutch (GD), Afro-Surinamese Dutch (ASD) and the Hindustani Surinamese-Dutch (HSD) communities in Amsterdam*.

The survey revealed that out of the 57 Ghanaian-Dutch respondents, 19 (33.3%) indicated that “Yes” they needed to receive more information about COVID-19 (Figure 1). In addition, 12 (21%) indicated that they “maybe” be open to receive more information on COVID-19. However, the majority (45.7%) answered “No”, indicating they did not need any more information. Equally, among the 54 Afro Surinamese-Dutch respondents who were asked, “would you like to receive more information about COVID-19?” 15 (28%) answered “yes”, indicating their need for more information on COVID-19. A further 11 (20%) of respondents answered “maybe”, showing that they were open to receive at least some additional information on COVID-19. However, more than half, 28 (52%) answered “No”, indicating that they have already had enough information and did not need any extra information. Out of the 49 respondents from the Hindustani Surinamese-Dutch community in Amsterdam, 26 (53%) responded “yes”, indicating that they would need to receive more information on COVID-19. In contrast, 20 (41%) responded “No”, showing that they did not need more information on COVID-19. Three respondents (6%) indicated that “maybe” they would like to receive additional information on COVID-19. Our in-depth interviews revealed that some respondents who did not like to receive additional information on COVID-19 because they were “tired of this Corona” and therefore preferred not to know more. According to one of the Afro Surinamese-Dutch woman we spoke:

*What other information is there. Is the old story we keep hearing? They do not try to even update it any better. They will tell us to wash hands, 1.5 meter, wear mask … Are we not just tired of this Corona? When I hear Corona I put my TV off. It's too much…*.

##### Kind of Information Respondents Needed

As listed in Table 2, quite a high proportion of respondents among the Ghanaian-Dutch respondents wanted to know more about the COVID-19 vaccination and the possible side effects. Most of them expressed their fear about the possible negative impact of the vaccine on their overall health especially when they described themselves as obese/overweight or had chronic diseases such as hypertension and diabetes. To some, they wanted to be vaccinated and needed to know when they might receive a letter or a call for appointment to get vaccinated. It was also common for people to inquire further about the different vaccines available and which had minor or no side effects.

**Table 2:** Top five (5) kinds of information on COVID-19 needed by respondents from the 3 selected communities.

*Ghanaian-Dutch respondents n = 57*	
1	Impact of the vaccine on their overall health
2	When they will receive a letter or a call for appointment to get vaccinated
3	Types of COVID-19 vaccines and their efficacy
4	COVID-19 vaccination and the possible side effects
5	Explanation on how the vaccine works and scientific data that supports it
*Afro Surinamese-Dutch n = 53*	
1	COVID-19 Vaccine, it's efficacy and side effects
2	How to protect oneself against COVID-19
3	Everything about COVID-19
4	The different kinds of COVID-19 vaccines and how to choose
5	COVID-19 variants and its significance
*Hindustani Surinamese-Dutch n = 47*	
1	Prevention/protection practices without vaccination
2	COVID-19 vaccination and possible side effects
3	Types of COVID-19 vaccines and their efficacy
4	COVID-19 variants and its implication of health
5	Post COVID-19 recovery and health effects

Based on individual responses from the Afro Surinamese-Dutch community, information on the COVID-19 vaccine, its efficacy and side effects, how to protect oneself against COVID-19, and the different kinds of COVID-19 vaccines and how to choose were mostly stated. The issue about the variants of COVID 19 was a concern to many participants, the different vaccines available and which had minor or no side effects, explanation on how the vaccine works and scientific data that supports it, how the coronavirus spreads and how to acquire immunity, measures related to education particularly for students, and when the COVID-19 pandemic will end. Only a few respondents needed to know everything about COVID-19.

In the Hindustani Surinames-Dutch community, a major proportion of the respondents wanted to know more about the COVID-19 vaccination and the possible side effects. Most of them expressed their fear about the possible negative impact of the vaccine on their overall health. There appear to be so much news on the new COVID-19 variants and how fast they spread. However, some respondents wanted to know the implication of contracting any of the new variants of the virus and whether it could have more debilitating effects on their health. A section of the respondents also wanted to receive information on alternative ways to deal or protect themselves from COVID-19 without taking the vaccination.

#### Respondents' Perceived Community Information Needs

Many of the respondents 29 (50.9%) out of 57 from the Ghanaian-Dutch community, indicated “Yes” that people in their community needed more information on COVID-19. Out of 53 respondents from the Afro Surinamese-Dutch community in Amsterdam, majority proportion, 37 (70%) were of the view that perhaps people within their community would need to receive more information on COVID-19 while among the 47 Hindustani Surinamese-Dutch respondents, 16 (34%) answered “Yes”, that people in their community needed more information on COVID-19. The top five kinds of information respondents from each community mentioned are listed in Table 3.

**Table 3:** The list of top 5 kinds of information perceived by respondents to be needed by their respective communities.

*Ghanaian-Dutch respondents n = 57*	
1	COVID-19 Vaccine, its efficacy and side effects
2	Explanation on how the vaccine works and scientific data that supports it
3	Types of COVID-19 vaccines and their efficacy
4	COVID-19 vaccination and the possible side effects
5	Everything about COVID-19
*Afro Surinamese-Dutch n = 53*	
1	Uncertainties/Theories about the existence of COVID-19
2	The need to adhere to the COVID-19 safety and prevention protocols
3	Everything about COVID-19
4	The influence of the views of family and friends on COVID-19
5	COVID-19 variants and its significance
*Hindustani Surinamese-Dutch n = 47*	
1	COVID-19 vaccine and its effects
2	Types of COVID-19 vaccines and the scientific reports on their efficacy
3	COVID-19 variants and its implication on health
4	Updates of every information on COVID-19
5	Prevention/protection without vaccination

Some respondents noted that more information about the COVID-19 vaccination, the types of vaccines being used at the “Prik locaties in Amsterdam”, scientifically proven information and efficacy of the vaccines, and how people could achieve immunity or protection from COVID-19 without the vaccines. Some respondents indicated that some people in their community appeared not updated about the current happenings on COVID-19, and so they may need updates on all the general information on COVID-19.

### Sources of Information on COVID-19

Among the respondents from the Ghanaian-Dutch community, TV/Radio programmes (both international and Netherlands-based news) were the most referred to source of information 20 (35.9%). followed by the dependence on Social media (Whatsapp, Facebook, etc) 7 (12.3%) as shown in Table 4. Among the Afro Surinamese-Dutch community, the Dutch TV/radio programmes 14 (27%) and the use of internet/search engine portals 14 (27%) were mostly used as sources of information. The dependence on family and friends 7 (14%) and Social media platforms 7 (14%) were the joint second most used sources of information on COVID-19. Only a single respondent (2%) relied on Newspapers/Flyers/Brochures. The Hindustani Surinamese-Dutch community in Amsterdam appeared to be highly dependent on family and friends 13 (26%) as the primary source of information on COVID-19 followed by the TV/Radio programmes 10 (21%), the internet/website search engine portals 9 (19%) and Social media 7 (15%).

**Table 4:** The primary sources used by respondents from the three communities to obtain information on COVID-19.

**Sources of information used by Respondents**	**GD**	**ASD**	**HD**	**Total (%)**
N	56	51	47	154 (100)
TV/Radio programme	20	14	10	44 (28.6)
Social media (whatsapp, facebook, etc)	7	7	7	21 (13.6)
Family and friends	8	7	12	27 (17.5)
Search engines/internet	3	14	4	21 (13.6)
website of GGD Amsterdam/RIVM	11	4	9	24 (15.6)
Emails/text/calls	1	0	0	1 (0.6)
Huisarts/ health workers	1	0	1	2 (1.3)
Newspapers/Flyers/Brochures	0	1	0	1 (0.6)
Others	5	4	4	13 (8.4)

During an in-depth interview with some of the respondents on why they preferred certain source of information, one of the Ghanaian-Dutch man, explained that “information is now everywhere” but he was careful what information to rely on and he preferred to listen to Dutch News portals because

*I live here now and I have to really understand what is happening here. Aside news, when I need very specific information I just go to the RIVM website and I get the most reliable and updated information*.

Unlike the Afro and Hindustani Surinamese-Dutch respondents who understand and speak the Dutch language, many respondents from the Ghanaian-Dutch community had inadequate understanding of the Dutch language. Despite the situation, some of the respondents from the Ghanaian-Dutch community noted that they always preferred to listen to Dutch news with their children or asked their adult or teenage children to explain to them what was said in the news when they did not fully understand. Some respondents also noted that some individuals within the community took up the responsibility to translate all the broadcast from the news and press conferences by the Dutch prime minister or minister of health into common ethnic-matched languages and shared on the social media platforms. These audio or video recordings were targeted to people within the communities that did not fully understand the Dutch language. Some respondents from the three ethnic communities expressed their appreciation for these interventions and patronized such sources of information as “the language was deemed simple, relatable, and comes home”. In this context, we observed from our fieldwork that simple language and being able to easily understand the information is important to many people within these communities.

However, in some cases the translations of such important information from Dutch to other languages have been received with less attention. According to a male respondent from the Hindustani Surinamese-Dutch “some social media explanations provided only a little information and in such situation certain vital elaborations are missed”. The vice-versa situation is when original messages are overly explained to loose the actual content. It is therefore important for translations to be accurate, clear and timely. In view of this, people from the Ghanaian-Dutch, Afro and Hindustani Surinamese-Dutch communities make efforts to search for specific information that they can relate with and apply to their situation. It is one thing to get the message about COVID-19 out, but more important is to get that message right through reachable sources and within time.

#### Age-Dependent Variation to Sources of Information Use

Age-dependent analysis of the data indicated that international news portals were the most preferred source of information on COVID-19 among respondents aged 35 years and above. In addition to this, Dutch news portals were the most popular among respondents aged 18 to 35 years, but remained the most preferred among respondents aged 56 years and above. Religious meetings, family and friends and social media were the most popular sources of information on COVID-19 among respondents aged 46 to 55 years. More so, the use of social media for information on COVID-19 was widely common among respondents aged 35 to 55 years old. Generally, the results also indicated that a majority of the total respondents relied highly on their preferred sources of information because of the tendency to provide them with authentic reports (58.9%) followed by readily available information (23.9%) and the simplicity of the language used (21.13%) and easy of understanding (14.08%).

## Discussions

Some research works show that the coronavirus pandemic generated a lot of media attention and education on COVID-19 control and prevention measures globally (28, 29). These education and media attention were often geared to reveal public perceptions and experience about the pandemic, and also identify factors that hamper or support efforts to curb global spread of the disease (28). Some study findings identified the need for widespread, and continuous public health education about the virus and COVID-19, especially among certain populations (30, 31). This was mainly because the knowledge level was perceived to be low among people from certain minority ethnic groups, those who had low education and low-income levels, black women and the unemployed (30, 31). In the Netherlands, the assumption from public discourse shows that people from minority ethnic groups have poor knowledge, or lack sufficient understanding of their attitudinal and behavioral risks to the coronavirus (32). However, this remains an assumption as this study revealed that majority of the respondents from the Ghanaian- and Surinamese-Dutch demonstrated good knowledge on the modes of transmission and prevention or mitigating measures about COVID-19.

As discussed in an earlier study (33), knowledge level on COVID-19 is linked directly to preventive behaviors that are important to reduce COVID-19 spread within Ghanaian-Dutch communities in the Netherlands. In this present study, our findings showed that a little over a third of the respondents (36.4%) demonstrated low behavioral risk of contracting COVID-19 as they indicated that they avoid or rarely used the public transport, or attended public and social events i.e., the church, mosque, supermarket, public transport, visit friends and family, among others. More than half (54.5%) had a moderate behavioral risk of contracting COVID-19 as they often or sometimes visited some of the locations noted earlier. These findings suggest that majority of people from the Ghanaian-Dutch, Afro Surinamese-Dutch and Hindustani Surinamese-Dutch appear to translate their good knowledge on COVID-19 into good preventive behaviors (i.e., low to moderate behavioral risk of contracting COVID-19). However, about 9.1% of the respondents showed high-risk behavior, as they were always present at public and social events. and reported that they had difficulty following some specific measures such as wearing a face/nose mask, avoiding visits to friends and family, and maintaining an interpersonal physical distance of 1.5 m. Regarding visiting friends and family, interestingly, many respondents in this present study particularly from the Ghanaian-Dutch community, found it difficult to turn down an invitation from family and close friends. This is not only because they were worried about maintaining these closer relationships, but also because, respondents perceived that their family and friends were less likely to contract COVID-19. These findings underscore the importance of social impressions, as people are concerned about what others think of them, and prioritize their views than what they must do.

Earlier studies have shown that unfavorable behaviors toward COVID-19 prevention are exacerbated by people's work or job category (34). In this present study, majority of the respondents were classified as essential workers, particularly healthcare and that the nature of their jobs made it difficult to maintain 1.5 m distances from their clients, patients and work colleagues. The findings in this study appear to reinforce the plight of essential workers (34, 35). It is therefore important for adequate measures to be taken to protect essential workers from risks linked to their job or work. From this perspective, it becomes imperative to distinguish between persons whose behavior increases exposure to contracting COVID-19 based on their occupation or in relation to close relations.

### Is This a Sign of Information Fatigue or a Lack of Information?

Global attempts to promote knowledge about the COVID-19 pandemic led to the ubiquity of health-related information across all media platforms. However, recent research suggests that “abundant” accessibility of information on COVID-19 could lead to adverse psychological effects, including anxiety, panic-based hoarding, and other unhealthy behaviors (28). Some of these consequences have been explained with the notion of information fatigue or overload (28, 29). Our findings highlight that many of the respondents who said they did not need to get more information on COVID-19 were concerned about getting “old” information. They felt that it was the same recycled information that they kept receiving and therefore did not see the need to get more. Other respondents also felt “tired of this Corona” and preferred not to know more.

This observed lack of interest has been shown to either influence or obscure the successful uptake and utilization of the information to either change or improve their behavioral choices regardless of the amount of information disseminated (36). As suggested in a recent study in Germany by Skulmowski and Standl (37), individual organizations interested in keeping people informed concerning COVID-19 should consider the use of personalized information strategies that avoid inducing negative emotional states. This present study by extension suggests that for minority ethnic groups, it is essential for surveys to be conducted on periodic intervals or employ digital innovations to ascertain specific information the people would need and engage with.

Alternatively, since this study was conducted during the second wave amid strict lockdown measures, many of the respondents had assimilated a lot of information on COVID-19. During the initial outbreak, many respondents were occupied with arming themselves with every bit of information they could find or help them stay safe. As a result most of the information that were shared on the television and other media platforms were things they already know about and so did not see the need to know more. A lot of the restrictions and regulations had become a second nature or habit of high proportion of our respondents and thus they felt the information was old. Thus, a reason for rejection for more information on COVID-19 became a natural consequence. Another explanation suggested by other researchers show that COVID-19 information fatigue had developed and made the public less interested in news surrounding the issue due to the ubiquity of the same information concerning COVID-19 (37, 38). The public may have become disinterested of this topic, at least in the sense that no active search for information is perceived to be necessary.

More so, some respondents did not want to receive more information on COVID-19 because of the persistence focus by the media on “bad news” that is, total number of persons who have contracted or died from the disease. The COVID-19 pandemic has been associated with pain, anxiety, depression, and loneliness among other mental health issues in the Netherlands (39, 40). According to some research findings, the repetition, relative abundance of recycled news and ubiquity of COVID-19 information particularly those that invoke worrying bad memories or health-related issues could lead people into a state of anger and also desensitized others (37, 41). This present study revealed that some respondents who did not need more information appeared to be more open to know more about how to “encourage and lift people up”.

While certain category of respondents did not seek additional information about COVID-19 public health-related topics and the negative effects of the pandemic, a large proportion wanted to know more about specific concerns. In this study, a many respondents across the three study communities wanted to know more about the COVID-19 vaccination and the possible side effects. Most of them particularly from Hindustani Surinamese-Dutch and Ghanaian-Dutch communities described themselves as obese/overweight or had chronic diseases such as hypertension and diabetes and feared the possible negative implication for their overall health and wellbeing. As uncertainties and misinformation appear to proliferate during this pandemic (42), directing communication efforts to specific populations, including those considered not at high risk such as younger adults could be beneficial to providing these groups with accurate and needed information.

At present, majority of the news have focused on the COVID-19 variants and how fast they spread. To some respondents, beyond the fast spread of the new COVID-19 variants they did not understand the implication of contracting any of the new variants of the virus. As the world health organization is working with researchers, health officials and scientists to understand what impact the new variants of the COVID*-*19 virus have on vaccines, a lot of respondents were waiting to decide their next actions toward COVID-19. Preliminary analyses have showed that the South Africa variant (501Y.V2) was associated with in-hospital mortality that was 20% higher in the second wave in South Africa than in the first wave (43). This finding was due mainly to the greater transmissibility of this variant, which rapidly overburdened health services and thus compromised timely access to hospital care and the quality of that care. The UK variant (B.1.1.7) has also been shown to be associated with a higher risk of death (44). A lot still remain unknown about the Delta and omicron variants among others beyond the greater transmissibility of this variant.

A section of the respondents also wanted to receive information on alternative ways to deal or protect themselves from COVID-19 without taking the vaccine. There is widespread acceptance of COVID-19 vaccines as the major breakthrough toward the fight and rapid eradication of the coronavirus disease (45). As we showed in an earlier publication (27), out of the total of 55 Ghanaian-Dutch respondents only 2 (3.6%) had taken vaccine and nearly half (47%) of the respondents indicated their readiness to take the COVID-19. Only 7 (13%) out of the 54 respondents from the Afro Surinamese-Dutch had already received the COVID-19 while nearly half 26 (49%) of the respondents indicated their willingness get the vaccine. Also among the Hindustani Surinamese-Dutch respondents, only 7 (14%) of the respondents had received the vaccine with nearly a half (47%) also willing to take the vaccine (27). A large proportion of respondents were not interested in taking the vaccine among the minority ethnic groups and this finding is consistent with earlier publication indicating about 40% of the Dutch population opposed the COVID-19 vaccine (46). As the findings of this present study shows that achieving a high uptake of the COVID-19 vaccine will be a challenge (45) and some respondents only wanted to receive information on alternative ways to deal or protect themselves from COVID-19 without taking the vaccine.

### Diverse Platforms Required for COVID-19 Education

The findings from this study showed that people from different communities use diverse ways to access information on COVID-19, and these various sources of information had various impacts on how people adhere to the COVID-19. The news around COVID-19 keeps changing rapidly especially with new data on research and there seem to be a lot of (mis)information also shared. This shows that if misinformation spreads across friends and family people could take that information as the truth.

Our results show that substantial misinformation and uncertainty about the virus and COVID-19 existed at the time of the conduct of this study particularly about vulnerable or most affected groups, transmission and risk-reduction strategies. From the point of view of our respondents, there appeared to be some uncertainties or theories about the existence of COVID-19. Some respondents feared their age, and the presence of other health conditions made them more vulnerable and that they could easily succumb to COVID-19. Earlier research works showed that elderly people who contracted COVID-19 were more likely to develop severe manifestations of the disease (47). However, accumulating evidence shows that there is the need to distinct between healthy aging and aging with frequent occurrence of multiple comorbidities (48). The suggestion is that although age is one of the major risk factors for COVID-19, most of the complications from COVID-19 arise out of the pre-existing health conditions. It is therefore very important for education to be intensified to draw a clear distinction so that the impression is not created that old age is synonymous to death when one contracts COVID-19.

The findings of this study demonstrate the information needs, knowledge level, perception and misconception about the COVID-19 pandemic among some minority ethnic groups in the Netherlands. This calls for additional education and training in public health preparedness in the Ghanaian-Dutch, Afro Surinamese-Dutch and Hindustani Surinamese-Dutch communities in Amsterdam. Besides improving the knowledge, attitude and preventive behaviors toward COVID-19 among minority ethnic groups, the respondents' feedback about the current pandemic can help policy makers and management taskforces to further align public education for effective outcomes in the these three communities in the Netherlands.

The major limitation of this study was that data collection was conducted at the period when the COVID-19 infection rate in the Netherlands had reached its second peak (second wave) admist strict lockdown that made it difficult to recruit a high number of study participants. In addition, the views expressed by individuals do not represent the collective or generalized view of the entire study population as only 33 out of the 160 respondents participated in the in-depth interviews. Although we provide timely and relevant data, there are certain limitations to this study. Our survey was time specific and as we can see from on-going developments, there is the need for further research to understand the changing dynamics regarding information needs of diverse group of people. Further research may explore the changing roles in mobilizing stakeholders to employ data-driven interventions in the management of pandemics.

## Conclusion

The COVID-19 pandemic continues to have a devasting effect, but there have been relaxations of COVID-19 regulations in many countries, which comes with several questions from people to know what this means for their wellbeing and that of others. In this study, respondents showed an overall good knowledge and perception of COVID-19; however, there appears to be a low level of compliance or adherence to the COVID-19 prevention and safety measures. In as much as there was lockdown during collection of data for this study, it hardly deterred people from still gathering together in large numbers. More so, the study area as indicated above had many shops and so while only essential stores were open there were still many market activities at the period. Therefore, there is a need to encourage and remind people to follow the preventive protocols and disseminate appropriate information timely to safeguard the safety of vulnerable people's lives. A high proportion of the respondents underscored how the COVID-19 pandemic had affected their new sense of awareness and responsibility to keep themselves and their close relations safe. These respondents raised concerns about the persistent circulation of “bad news” on COVID-19 to the neglect of the flourishing or “good news”. Many thus advocated for messages that provide encouragement and hope that collective action and responsibility could go a long way to alleviate some of the negative consequences of the COVID-19 pandemic. The reasons behind why some respondents felt there was bad news in the media, however, there are on-going fieldwork and data collection to analyse how respondents felt and the full reasons behind that.

As our data showed the impact of the COVID-19 pandemic on public health requires a global bioethical reflections and responses as discussed in the recently published special issue on “Ethics and COVID-19: The Bioethics of a ‘Job Well Done’ in Public Health” by the Frontiers Health services journal. People's information choices go beyond the individual and incorporate ideas of health with social factors, based on a more relational approach (49). Our findings highlight how the COVID-19 pandemic has revealed the reality of policy gaps and information gaps that people from minority ethnic groups are confronted with and its effect on their well-being. Many people's concerns arise from inadequate information or disinformation or not knowing where to get the right and accurate information. Given this, policymakers may need to tailor efforts into regular updates and resources that provide complete answers to the concerns that arise from time to time. Specifically to COVID-19 vaccination, information dissemination and the safety or efficacy and benefits of the COVID19 vaccination are necessary. Specific focus should be the importance and efficacy of vaccines, and messages should counteract and dispel erroneous previously held views on vaccines.Therefore, it is crucial to scale up the community's awareness of COVID-19 prevention, testing, vaccination, and mitigation strategies through appropriate media outlets.

As our research shows, information and education programs on dispelling myths and fact-checking conflicting information on COVID-19 continue to evolve. Subsequent research may need to focus on how after a proportionally high number of vaccinations, people perceive risk and seek information. We suggest that there is the need to look at how health officials, government authorities, and religious leaders among others make a concerted and cohesive effort in information dissemination taking into consideration changing times.

## Data Availability Statement

The raw data supporting the conclusions of this article will be made available by the authors, without undue reservation.

## Ethics Statement

The studies involving human participants were reviewed and approved by AISSR Ethics Board. Written informed consent for participation was not required for this study in accordance with the national legislation and the institutional requirements.

## Author Contributions

AZB and DA-B conceived and planned the study, methodology and execution, supervised, carried out the data collection, verified the analytical methods, discussed the results, and contributed to the final manuscript. Both authors contributed to the article and approved the submitted version.

## Conflict of Interest

The authors declare that the research was conducted in the absence of any commercial or financial relationships that could be construed as a potential conflict of interest.

## Publisher's Note

All claims expressed in this article are solely those of the authors and do not necessarily represent those of their affiliated organizations, or those of the publisher, the editors and the reviewers. Any product that may be evaluated in this article, or claim that may be made by its manufacturer, is not guaranteed or endorsed by the publisher.

## References

[1] Imai N Dorigatti I Cori A Donnelly C Riley S Ferguson N. Report 2: Estimating the Potential Total Number of Novel Coronavirus Cases in Wuhan City, China. London: Imperial College London (2020).

[2] Gates B. Responding to Covid-19 — A once-in-a-century pandemic? N Engl J Med. (2020) 382:1677–9. 10.1056/NEJMp2003762 32109012

[3] World Health Organisation. Coronavirus disease (COVID-19) Weekly Epidemiological Update and Weekly Operational Update March/April 2021. (2021). Available online at: https://www.who.int/emergencies/diseases/novel-coronavirus-2019/situation-reports. (accessed July 6, 2021).

[4] World Health Organisation. COVID-19 Strategy Update 14th April 2020. (2020). Available online at: https://www.who.int/docs/default-source/coronaviruse/covid-strategy-update-14april2020.pdf (accessed July 6, 2021).

[5] RIVM Rijksinstituut voor Volksgezondheid en Milieu,. (2021). Committed to Health and Sustainability, Current Information About COVID-19. Available online at: https://www.rivm.nl/en/coronavirus-covid-19/current-information (accessed July 14, 2021).

[6] Oxford COVID-19 Government Response Tracker. Government Response Tracker Now Includes Data on Vaccination Policies Around the World. (2021). Available online at: https://www.bsg.ox.ac.uk/news/government-response-tracker-now-includes-data-vaccination-policies-around-world (accessed December 21, 2021).

[7] Cao X. COVID-19: immunopathology and its implications for therapy. Nat Rev Immunol. (2020) 20:269–70. 10.1038/s41577-020-0308-3 32273594 PMC7143200

[8] Khadka S Hashmi FK Usman M. Preventing COVID-19 in low-and middle-income countries. Drugs Ther Perspect. (2020) 36:250–2. 10.1007/s40267-020-00728-8 32292266 PMC7152742

[9] Asnakew Z Asrese K Andualem M. Community risk perception and compliance with preventive measures for COVID-19 pandemic in Ethiopia. Risk Manag Healthc Policy. (2020) 13:2887–97. 10.2147/RMHP.S279907 33335434 PMC7737628

[10] Coyer L Boyd A Schinkel J Agyemang C Galenkamp H Koopman AD. SARS-CoV-2 antibody prevalence and determinants of six ethnic groups living in Amsterdam, the Netherlands: a population-based cross-sectional study, June-October 2020. medRxiv. (2021) 03.08.21252788. 10.1101/2021.03.08.21252788 PMC8739540 34992110

[11] Amgain K Neupane S Panthi L Thapaliya P. Myths versus truths regarding the novel coronavirus disease (COVID-2019) outbreak. J Karnali Acad Health Sci. (2020) 3:1–6. 10.3126/jkahs.v3i1.28367

[12] Oliver RM. What makes young people think positively about social distancing during the corona crisis in Germany? Front Sociol. (2020) 5:61. 10.3389/fsoc.2020.00061 33869467 PMC8022463

[13] Bodner J Welch W Brodie I. COVID-19 Conspiracy Theories: QAnon, 5G, the New World Order and Other Viral Ideas. Jefferson: McFarland (2020).

[14] Grimes DR. Medical disinformation and the unviable nature of COVID-19 conspiracy theories. PLoS ONE. (2021) 16:e0245900. 10.1371/journal.pone.0245900 33711025 PMC7954317

[15] Morgan M. Why meaning-making matters: the case of the UK Government's COVID-19 response. Am J Cult Sociol. (2020) 8:270–323. 10.1057/s41290-020-00121-y 33078075 PMC7557151

[16] Worldometer. COVID-19 CORONAVIRUS PANDEMIC: Cases, Deaths and Recovered. (2021). Available online at: https://www.worldometers.info/coronavirus/ (accessed on July 14, 2021).

[17] de Boer H. COVID-19 in Dutch higher education. Stud High Educ. (2021) 46:1; 96–106, 10.1080/03075079.2020.1859684

[18] NL Time. Poorer Neighborhoods Hit Harder by Covid-19 Infections: Report. Monday, October 12, 2020. (2020). Available online at: https://nltimes.nl/2020/10/12/poorer-neighborhoods-hit-harder-covid-19-infections-report (accessed November 1, 2021).

[19] Reiter PL Mira LK. Racial/ethnic differences in knowledge, attitudes, and beliefs about COVID-19 among adults in the United States. Front Public Health. (2021) 9:653498. 10.3389/fpubh.2021.653498 34046389 PMC8144327

[20] SEO Economisch Onderzoek. Amsterdam, Netherlands: Self-Evaluation Report. OECD Reviews of Higher Education in Regional and City Development. Amsterdam: IMHE (2009).

[21] CBS, Centraal Bureau voor de Statistiek. Statistics Netherlands. StatLine – Bevolking; leeftijd, herkomstgroepering, geslacht en regio. Kralendijk: CBS (2018).

[22] CBS, Centraal Bureau voor de Statistiek. Statistics Netherlands. StatLine – Bevolking; leeftijd, herkomstgroepering, geslacht en regio. Kralendijk: CBS (2021).

[23] Van der Veer P Vertovec S. Brahmanism abroad: on caribbean hinduism as an ethnic religion. Ethnology. (1991) 30:149–66. 10.2307/3773407

[24] Bakuri A Spronk R van Dijk R. Labour of love: Secrecy and kinship among Ghanaian-Dutch and Somali-Dutch in The Netherlands. Ethnography. (2020) 21:394–412. 10.1177/1466138120938808

[25] Bakuri AZ. In Pursuit of Well-Being: Sexuality and Religion Among Ghanaian-Dutch and Somali-Dutch in the Netherlands. PhD Dissertation. Amsterdam: University of Amsterdam, The Netherlands (2021).

[26] Azene ZN Merid MW Muluneh AG Geberu DM Kassa GM Yenit MK. Adherence towards COVID-19 mitigation measures and its associated factors among Gondar City residents: a community-based cross-sectional study in Northwest Ethiopia. PLoS ONE. (2020) 15:e0244265. 10.1371/journal.pone.0244265 33378332 PMC7773181

[27] Antwi-Berko D Bakuri AZ Otabil KB. Kwarteng A. Determinants and variations of COVID-19 vaccine uptake and responses among minority ethnic groups in Amsterdam, the Netherlands. Front Public Health. (2022) 10:761987. 10.3389/fpubh.2022.761987 35252081 PMC8891150

[28] Oyebode O Ndulue C Adib A Mulchandani D Suruliraj B Orji FA. Health, psychosocial, and social issues emanating from the COVID-19 pandemic based on social media comments: text mining and thematic analysis approach. JMIR Med Inform. (2021) 9:e22734. 10.2196/22734 33684052 PMC8025920

[29] Williamson B Eynon R Potter J. Pandemic politics, pedagogies and practices: digital technologies and distance education during the coronavirus emergency. Learn Media Technol. (2020) 45:2, 107–14, 10.1080/17439884.2020.1761641

[30] Ali S Foreman J Tozan Y Capasso A Jones A DiClemente R. Trends and predictors of COVID-19 information sources and their relationship with knowledge and beliefs related to the pandemic: nationwide cross-sectional study. JMIR Public Health Surveill. (2020) 6:e21071 10.2196/21071 32936775 PMC7546863

[31] McCormack LA Squiers L Frasier AM Bevc C Lynch M Bann CM. Gaps in Knowledge About COVID-19 Among US Residents Early in the Outbreak. Public Health Rep. (2021) 136:107–16. 10.1177/0033354920970182 33176108 PMC7856374

[32] Van der Leun JP van der Woude MAH. Ethnic profiling in the Netherlands? A reflection on expanding preventive powers, ethnic profiling and a changing social and political context. Pol Soc. (2011) 21:4, 444–55. 10.1080/10439463.2011.610194

[33] Antwi-Berko D Bakuri AZ. Adherence to COVID-19 Preventive Measures Among the Ghanaian-Dutch Community in the Netherlands; a Mixed Method Study in Amsterdam. (2021). Available online at: 10.2139/ssrn.3907843 (accessed August 1, 2021).

[34] The Lancet. The plight of essential workers during the COVID-19 pandemic. Lancet. (2020) 395:1587. 10.1016/S0140-6736(20)31200-9 32446399 PMC7241973

[35] Guerrero LR Avgar AC Phillips E Sterling MR. They are essential workers now, and should continue to be: social workers and home health care workers during COVID-19 and Beyond. J Gerontolog Soc Work. (2020) 63:6–7, 574–6. 10.1080/01634372.2020.1779162 32543355 PMC7738393

[36] Griffin RJ Dunwoody S Yang ZJ. Linking risk messages to information seeking and processing. Ann Int Comm Assoc. (2016) 36:323–62. 10.1080/23808985.2013.11679138

[37] Skulmowski A Standl B. COVID-19 information fatigue? A case study of a German university website during two waves of the pandemic. Hum Behav Emerg Tech. (2021) 3:350–356. 10.1002/hbe2.260 34222832 PMC8239648

[38] Koh PKK Chan LL Tan EK. Messaging fatigue and desensitisation to information during pandemic. Arch Med Res. (2020) 51:716–7. 10.1016/j.arcmed.2020.06.014 32713728 PMC7377807

[39] Luijten MAJ van Muilekom MM Teela L Polderman TJC Terwee CB Zijlmans J. The impact of lockdown during the COVID-19 pandemic on mental and social health of children and adolescents. Qual Life Res. (2021) 15:1–10. 10.1101/2020.11.02.20224667 33991278 PMC8122188

[40] van Tilburg TG Steinmetz S Stolte E van der Roest H de Vries DH. Loneliness and mental health during the COVID-19 pandemic: A study among Dutch older adults. J Gerontol B Psychol Sci Soc Sci. (2020) 76:e249–55. 10.1093/geronb/gbaa111 32756931 PMC7454922

[41] Baseman JG Revere D Painter I Toyoji M Thiede H Duchin J. Public health communications and alert fatigue. BMC Health Ser Res. (2013) 13:295. 10.1186/1472-6963-13-295 23915324 PMC3751004

[42] Kim HK Ahn J Atkinson L Kahlor LA. Effects of COVID-19 misinformation on information seeking, avoidance, and processing: a multicountry comparative study. Sci Commun. (2020) 42:586–615. 10.1177/1075547020959670 PMC7492825 38603002

[43] Pearson CAB Russell TW Davies N Kucharski AJ CMMID COVID-19 working group Edmunds WJ. Estimates of Severity and Transmissibility of Novel SARS-CoV-2 Variant 501Y.V2 in South Africa. London: CMMID Repository (2021).

[44] Horby P Huntley C Davies N Edmunds J Ferguson N Medley G Semple C. NERVTAG Paper on COVID-19 Variant of Concern B.1.1.7. London: Department of Health and Social Care, Scientific Advisory Group for Emergencies (2021).

[45] Loomba S de Figueiredo A Piatek SJ de Graaf K Larson HJ. Measuring the impact of COVID-19 vaccine misinformation on vaccination intent in the UK and USA. Nat Hum Behav. (2021) 5:337–48. 10.1038/s41562-021-01056-1 33547453

[46] DutchNews.nl. Why Are so Many People in the Netherlands Opposed to a Covid Vaccine? (2020). Available online at: https://www.dutchnews.nl/news/2020/12/why-are-so-many-people-in-the-netherlandsopposed-to-a-covid-vaccine/ (accessed July, 15 2021).

[47] Liu Y Huang F Xu J Yang P Qin Y Cao M. Anti-hypertensive Angiotensin II receptor blockers associated to mitigation of disease severity in elderly COVID-19 patients. MedRxiv. (2020) 20039586. 10.1101/2020.03.20.20039586

[48] D'ascanio M Innammorato M Pasquariello L Pizzirusso D Guerrieri G Castelli S. Age is not the only risk factor in COVID-19: the role of comorbidities and of long staying in residential care homes. BMC Geriatr. (2021) 21:63. 10.1186/s12877-021-02013-3 33451296 PMC7809533

[49] Valera L López Barreda R. Bioethics and COVID-19: considering the social determinants of health. Front Med. (2022) 9:824791. 10.3389/fmed.2022.824791 35391891 PMC8980461

