# Supplementary material for: “What Other Information Is There?”: Identifying Information Gaps, Perceptions and Misconceptions on COVID-19 Among Minority Ethnic Groups in the Netherlands
Source: Front Health Serv. 2022 Jul 8;2:824591. doi: 10.3389/frhs.2022.824591 (PMC10012720; doi:10.3389/frhs.2022.824591)
Supplement: Supplementary file 1 [file Table_1.DOCX]

**PERCEPTIONS AND RESPONSES TO CORONAVIRUS DISEASE (COVID-19) AMONG GHANAIAN-DUTCH, AFRO AND HINDUSTANI SURINAMESE-DUTCH COMMUNITIES IN AMSTERDAM**

All personal information will remain anonymous and no personal or identifying information from this survey will be shared in any of our reports or publications.

**A. General Information**

Date of response: ____/___/2021

1. Age: ____________

2. Sex:

☐ Male

☐ Female

☐ Other

☐ Prefer not to answer

3. What is your race/ethnicity? (Select all that apply)

☐ African-Surinamese

☐ South-Asian Surinamese

☐ Surinamese-Dutch

☐ Dutch

☐ Other

4. Highest level of education:

☐ Doctoral degree

☐ Masters degree

☐ Bachelor’s degree

☐ Vocational or technical school

☐ Senior high school or equivalent

☐ Junior high school

☐ Primary or Elementary school

☐ None

5. In which area are you employed?

☐ Health care

☐ Education

☐ Transport

☐ Construction

☐ Hospitality and Catering

☐ Administrative/legal and social work

☐ Pastoral/religious office

☐ Sales person

☐ Security

☐ Domestic chores/Cleaning

☐ Student

☐ Unemployed

☐ Retired

☐ Prefer not to answer

☐ Other, Please specify______________________

6. Current employment status:

☐ Part-time

☐ Full-time

☐ Unemployed

☐ Retired

☐ Student

7. Source of income: ☐ salary

☐ self-employed

☐ Alimony

☐ family/friends support

☐ Government support (Social, Pension, etc)

☐ Other, Please specify ______________________

☐ Prefer not to answer

9. How many persons live with you in your home

☐ 1

☐ 2

☐ 3

☐ 4

☐ 5 or more

10. How many adults (**aged ≥18 years**) live with you in your home?

☐ 1

☐ 2

☐ 3

☐ 4

☐ 5 or more

11. Would you be willing to be contacted for a follow-up interview in future?

☐ Yes

☐ No

12. How would you prefer we contact you later for an in-depth interview?

☐ Telephone or mobile **→** Telephone number: ______________________

☐ Email **→**Email address: __________________________

☐ Home visit **→**Home address: __________________________

**B. Information needs on COVID-19**

1. Would you like to receive more information about COVID-19?

☐ Yes

☐ No

2. What kind of information on COVID-19 would you personally like to receive more about?

☐ Mode of transmission/how people contract COVID-19

☐ Signs/symptoms and Testing for COVID-19

☐ Treatment and health consequences of COVID-19

☐ Preventive measures/best ways to protect oneself and loved ones

☐ COVID-19 Vaccination/Possible side effects if any

☐ Other, Please specify

3. Do you think people in your community would also like to receive more information on COVID-19?

☐ Yes

☐ No

4. Which type of information on COVID-19 should be prioritised in the community?

☐ Mode of transmission/how people contract COVID-19

☐ Signs/symptoms and Testing for COVID-19

☐ Treatment and health consequences of COVID-19

☐ Preventive measures/best ways to protect oneself and loved ones

☐ COVID-19 Vaccination/Possible side effects if any

☐ Other, Please specify

5. How would you want this information of COVID-19 be delivered to you?

☐ Telephone call/text messages

☐ TV/Radio programmes

☐ Flyers/Brochures/Newspaper

☐ Use Information vans or centers

☐ GGD Amsterdam/RIVM websites

☐ Other, please specify

**B. Sources of Information on COVID-19**

Which of the following is/are your most reliable source of information regarding COVID-19?

☐ International news portal (CNN, BBC, Euronews, etc)

☐ Dutch news portal (NPO, RTL, etc)

☐ Ghanaian/Surinamese news portals

☐ Online news portal/search engines

☐ Public Health Services Amsterdam (GGD Amsterdam)

☐ Church/Mosque/religious meetings

☐ Family and friends

☐ Social media

☐ Other, please specify _____________

**C. Knowledge and Behavioural risk to COVID-19 (ALL participants)**

1. What are the likely places through which people contract COVID-19?

☐ Church or Mosque

☐ Workplace

☐ Home

☐ Attending funerals

☐ Attending weddings

☐ Public events (music festivals)

☐ Other social events (birthday parties, anniversary questions, etc)

☐ Restaurant/bar

☐ Public transport

☐ Supermarket/ Shops/market place

☐ GP/dentist practice/Pharmacy/other medical professional office

☐ Travel

☐ Other, please specify ­­­­­­­­­____________________________

2. How often do you visit any of places listed in Question 1?

☐ Always

☐ Sometimes

☐ Often

☐ Rarely

☐ Never

☐ I do not know

3. Why are people likely to contract COVID-19 from these places?

☐ Hugging/handshakes

☐ Failure to wash hands/use hand sanitizers

☐ Difficult to keep 1.5m distances

☐ Failure to wear face mask

☐ Failure to self-quarantine/isolate after travel

☐ Other, please specify ­­­­­­­­­____________________________

4. What are you likely to do when you experience symptoms similar to COVID-19?

☐ Call the GP

☐ Get tested for COVID-19

☐ Visit the pharmacist

☐ Stay home

☐ Do nothing

☐ Other, please specify ­­­­­­­­­____________________________

5. Which of the following COVID-19 preventive measures are aware of?

☐ Clean your hands often (use soap/water, or alcohol-based handrub)

☐ Maintain a safe distance (1.5m) from each other

☐ Avoid touching your eyes, nose or mouth

☐ Cover your nose and mouth when you cough or sneeze.

☐ Stay home if you feel unwell.

☐ Limit social gatherings and time spent in crowded places.

6. Which of the following do you find important for preventing COVID-19 infection? (Please options that apply)

☐ Wearing face and nose mask

☐ Hand washing and hygiene

☐ Maintaining 1.5m physical distance

☐ Cover mouth when sneezing or coughing

☐ Avoiding social and public gatherings

☐Avoiding visiting friends and families

☐ Reducing the number of visitors at home/office

☐ Staying home when having mild symptoms like sneezing and coughing

☐ Getting tested for COVID

☐ Other, please specify _____________

☐ None of the above

7. Which of the following COVID-19 prevention measures do you find difficult to follow or adhere to?

☐ Wearing face and nose mask

☐ Hand washing and hygiene

☐ Maintaining 1.5m physical distance

☐ Cover mouth when sneezing or coughing

☐ Avoiding social and public gatherings

☐Avoiding visiting friends and families

☐ Reducing the number of visitors at home/office

☐ Staying home when having mild symptoms like sneezing and coughing

☐ Getting tested for COVID

☐ Other, please specify _____________

☐ None of the above

8. Why do you find it challenging to adhere to the COVID-19 prevention measures?

☐ COVID-19 is not real

☐ I am a sociable person

☐ Do not like covering my make-up/face

☐ Wearing mask is uncomfortable

☐ Cost of mask is expensive

☐ Due to the nature of my job

☐ It is stressful/Time-consuming

☐ Other, please specify _____________

9. When do you find it a challenge to adhere to the COVID-19 prevention measures?

☐ Presence of friend/family

☐ Presence of work colleagues

☐ Presence of members of your religious group

☐ In the market

☐ Walking on the streets

☐ Other, please specify _____________

10. Which of the following is MOST likely to influence you to adhere the COVID-19 prevention measures?

☐ Increase education

☐ Use of COVID-19 ambassadors

☐ Use of religious leaders

☐ Its my responsibility

☐ Peer influence

☐ Fear of hospitalization or death

☐ Other, please specify _____________

*---THE END---*

*THANK YOU VERY MUCH*
